# Extrusion of the Distal Catheter From the Umbilicus: A Case Report of a Rare Complication After Ventriculoperitoneal Shunt and Its Management

**DOI:** 10.3389/fped.2020.00228

**Published:** 2020-05-27

**Authors:** Yi Xia, Fang He, Zhen Ren, Chao Wang

**Affiliations:** ^1^Department of Neurosurgery, Tangdu Hospital of the Fourth Military Medical University, Xi'an, China; ^2^Department of Outpatient, The 316th Military Hospital of China, Beijing, China; ^3^Department of Ultrasound, Xijing Hospital of the Fourth Military Medical University, Xi'an, China

**Keywords:** hydrocephalus, ventriculoperitoneal shunt, complications, extrusion, umbilicus

## Abstract

Spontaneous extrusion of the distal catheter from the umbilicus following a ventriculoperitoneal shunt (VPS) for the treatment of hydrocephalus is an extremely rare complication. Here, we describe an 8-years-old boy who underwent a VPS for communicating hydrocephalus and thereafter the distal part of the catheter was extruded through the umbilicus. The extrusive part of the peritoneal catheter was successfully cut off with a laparoscope, keeping the remaining catheter in place and functional. The subsequent recovery process was uneventful. To the best of our knowledge, there have been no reports of using laparoscopy to cut the protruding part and replace the shunt end. By keeping the function of the original shunt pipe, this case report offers an innovative and informative approach to treating this complication.

## Introduction

A ventriculoperitoneal shunt (VPS) is a common surgical intervention performed to treat different kinds of hydrocephalus in children and adults. However, postoperative complications such as infection, failure of a valve, catheter migration, obstruction of the shunt, and over-drainage are common (1). In contrast, extrusion of the peritoneal catheter from the umbilicus is rare. Here, we report such an unusual case.

## Case Presentation

An 8-years-old boy presented at our unit with a visible catheter extrusion through the umbilicus 6 months after VPS. For his past medical history, a hemispherectomy for intractable epilepsy had been conducted 1 year earlier. A computed tomography (CT) scan of the head 5 months after the operation showed communicating hydrocephalus. Subsequently, the child underwent a right-sited medium-pressure VPS using the Codman Hakim programmable valve system (Codman, Johnson Company, Raynham, USA) to relieve the hydrocephalus. The length of the intraperitoneal catheter was about 50 cm, which was chosen taking into account that the patient's height will increase with normal growth and development. The shunt system has an antisiphon valve but no antibacterial activity. The child presented with a fever with a daily axillary temperature ranging from 38 to 39°C 1 week after the VPS. At the same time, he had abdominal symptoms such as pain and tension in the abdominal muscles. The plain abdominal radiography showed intestinal tympanites.

Culture of cerebrospinal fluid (CSF) extracted via lumbar puncture grew methicillin-resistant *S. epidermidis*, and blood culture grew *S. simulans*. Consequently, central nervous system infection and septicemia were definitely diagnosed based on the above results. Intravenous vancomycin was administered based on the drug sensitivity test. He was discharged from the hospital after successful recovery.

**Figure 1:**
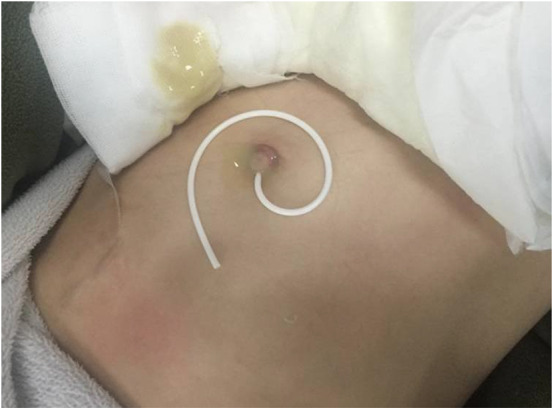
The distal catheter extruded from the umbilicus after VPS.

**Figure 2:**
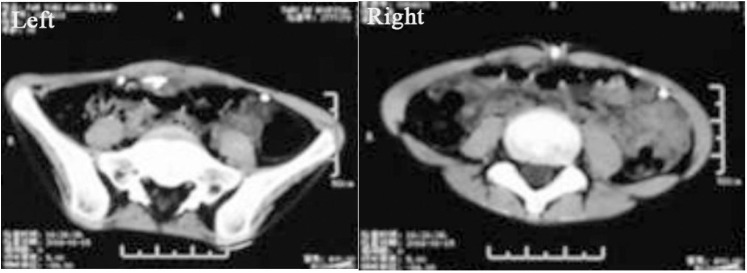
Abdominal axial CT (left) showing that there is fluid around the shunt at the superficial part of the abdomen around the umbilicus. CT (right) demonstrating that the shunt catheter was extruded through the umbilicus.

However, the umbilicus was red and swollen 6 months after VPS. The local skin ruptured, and yellow fluid was discharged from the fistula. The shunt catheter subsequently extruded through the fistula for about 25 cm length (Figure 1). Abdominal ultrasonography revealed that there was effusion under the umbilicus. Culture of the effusion via ultrasound-guided puncture grew methicillin-resistant *S. epidermidis*. Abdominal CT scan revealed that the effusion was encapsulated and localized around the umbilicus (Figure 2). The laboratory examinations of CSF and blood were normal. Most importantly, the patient's vital signs were stable.

**Figure 3:**
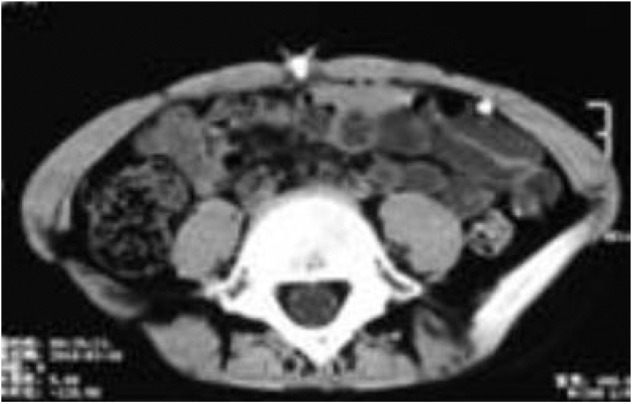
Abdominal CT revealing that the effusion disappeared.

**Figure 4:**
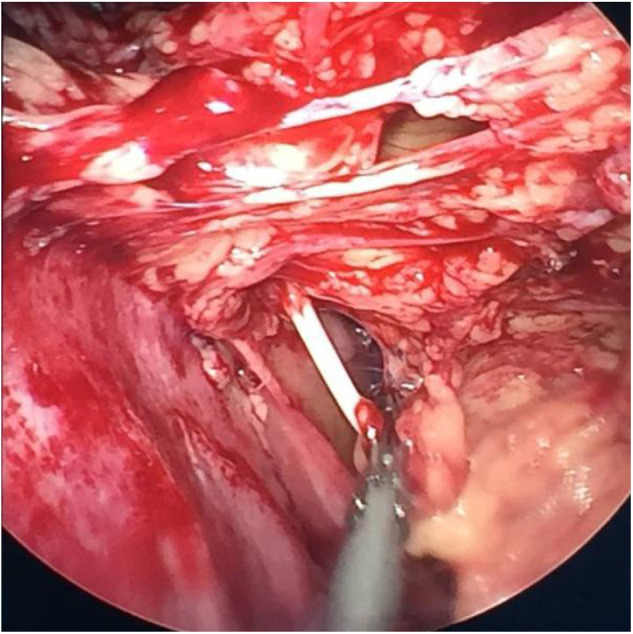
Extensive organ adhesions in the abdomen, including the digestive tract, greater omentum, and peritoneum, were visible.

**Figure 5:**
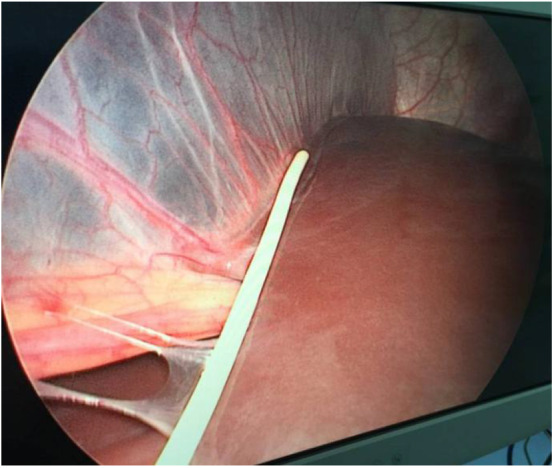
The extrusive part of the shunt was cut off, and the intra-abdominal part was repositioned on the diaphragm-facing surface of the liver using the laparoscope.

Based on these findings, we carried out the following treatments. First, the extrusive catheter was connected with a closed drainage bag for CSF collection. Secondly, according to the drug sensitivity test, vancomycin and cefoperazone sulbactam sodium were still used for 2 weeks to control local infection of the umbilicus. Thirdly, the fistula was washed with a disinfectant daily and drained smoothly. After a 2-weeks treatment, the patient showed no symptoms of infection or hydrocephalus. He was discharged with the extrusive catheter and followed up during dressing change at our outpatient clinic. Three months later, an abdominal CT revealed that the effusion had disappeared (Figure 3). Subsequently, the peritoneal catheter was revised via a laparoscopic procedure, but not totally replaced. During the operation, extensive adhesions of intra-abdominal organs, including the digestive tract, greater omentum, and peritoneum, were found. Connective tissue proliferation was also found to form a granular mass enclosing the fistula and catheter (Figure 4). Adhesiolysis was performed after laparoscopic exploration of the abdomen. Finally, the extrusive part of the shunt was cut off and the intra-abdominal part was repositioned on the diaphragm-facing surface of the liver after ensuring its patency, observing CSF dripping from its distal end (Figure 5). The child was discharged on the 7th day after surgery. The fistula of the umbilicus healed up. The plain abdominal radiograph and CT during the follow-up showed that the catheter was in place and well-functional.

## Discussion

VPS is the most common surgical therapy for the management of hydrocephalus of diverse etiologies. However, it has efficacy but does not guarantee absolute safety since complications are reported in some cases, especially the children, who survive long after VPS (2). A variety of abdominal complications have been reported with an acute abdomen, intestinal perforation, or obstruction, peritonitis, pseudocyst, and umbilical fistula (3–7). Here, we present a rare case that had the distal catheter extruded from the umbilicus after VPS. There were few previous reports of such a complication (8). The exact mechanism leading to this complication remains unknown.

A misbalance between the inflammatory reaction, either sterile or infectious, and body immunity is a possible reason (9).

In our case, it was traced back to 1 week after VPS; the cultures of CSF and blood were positive for bacteria, which indicated systemic infection after VPS. Abdominal infection had led to severe peritonitis and abdominal adhesions, which were confirmed by laparoscope. The inflammation was gradually localized to the umbilicus after the infection had been controlled. The distal catheter migrated to the umbilicus under the impulse of intestinal peristalsis. Then, the tip of the shunt was enclosed and fixed under the umbilicus by the proliferation of the connective tissue in the process of eliminating the abdominal infection. Nevertheless, the compounded fluid of CSF and effusion was continually collected and invaded the umbilicus, which is the weakest part of the abdominal wall. A fistula ultimately formed through the umbilicus. Finally, the catheter extruded from the fistula.

There are two different opinions on the management of this complication, either a revision or a replacement of the abdominal catheter. Based on our experience, we suggest that the abdominal shunt should be revised. If this method fails, the shunt should be replaced. This is the only approach that guarantees optimal cost-effectiveness. The abdomen has a very strong capacity to defend against inflammation, and it is not necessary to excessively worry about this complication worsening if the vital signs are stable. Even antibiotics may not be necessary to prevent an infection except in the perioperative period. In addition, keeping the effusion draining smoothly is very important.

Teegala and Kota reported two cases of ventriculoperitoneal shunt herniation through the anus (10). Malnutrition and infection were analyzed as possible causes. In most cases, intestinal perforation is asymptomatic. In a few cases, complications such as intestinal obstruction, adhesion, and intussusception can occur, which need to be treated as soon as possible. Similar to this case, there was no sepsis after herniation of the shunt tube. In cases of no perforating peritonitis or no abdominal abscess, there is often simply herniation from the umbilicus without other complications, and there is no need for a formal laparotomy (11–13). The patient was followed up for 6 months without any symptoms or complications (follow-up results were not included).

In this case, the exposed part of the shunt was cut off and removed through laparoscope, and the rest of the distal end of the shunt was fixed on the diaphragm side of liver. According to a large number of previous clinical reports, adhesive peritonitis is the main cause of the complications of the abdominal end. For infectious complications, the previous treatment methods include antibiotics and the removal of the shunt. Many doctors prefer to replace a new shunt after the removal of the old one to avoid the aggravation of hydrocephalus, but this greatly increases the pain and economic burden of patients.

In this case, we first drained the distal end of the shunt tube externally and carried out anti-infective treatment at the same time. The re-operation of VPS was not chosen for the following reasons. First, the child had developed a shunt tube dependency. If the shunt tube is removed directly, a lateral ventricle puncture is required to relieve hydrocephalus. After the infection of abdominal cavity is improved, which may take a long time, the re-operation of VPS can be performed. This strategy increases the risk of surgery and the possibility of intracranial infection. Then, during the 3-months drainage period, the dressings were changed in the outpatient clinic, and the patient was carefully nursed at home but not in the hospital. Furthermore, in terms of economic cost, the cost of re-operation of VPS is about 50,000 RMB in China, while the cost of laparoscopic surgery is about 10,000 RMB.

For the use of laparoscopy, in addition to reducing the chance of infection, the abdominal end can be fixed on the diaphragmatic surface of the liver, far away from the greater omentum, so as to reduce the probability of recurrence of adhesive peritonitis. This is also the difference between the present case and the previous reports.

## Conclusions

In summary, the extrusion of the abdominal shunt from an umbilical fistula is a serious complication because of abdominal infection, which is very rare. This report describes a pediatric case of an extrusion of the distal catheter from the umbilicus as a rare complication of VPS. This case report shares a relatively simple and effective therapeutic method, which is innovative and scientific to some extent. We hope to provide a simple and economic treatment option for neurosurgeons in the treatment of such complications.

For the use of laparoscopy, in addition to reducing the chance of infection, the abdominal end can be fixed on the diaphragmatic surface of the liver, far away from the greater omentum, so as to reduce the probability of recurrence of adhesive peritonitis.

## Data Availability Statement

All datasets generated for this study are included in the article/supplementary material.

## Ethics Statement

The studies involving human participants were reviewed and approved by Medical Ethics Committee of Tangdu Hospital, Fourth Military Medical University. Written informed consent to participate in this study was provided by the participants' legal guardian/next of kin. Written informed consent was obtained from the legal guardian for the publication of any potentially identifiable images or data included in this article. Informed consent was obtained from all individual participants included in the study.

## Author Contributions

YX and CW contributed conception and design of the study. ZR wrote the first draft of the manuscript. FH wrote sections of the manuscript. All authors contributed to manuscript revision, read, and approved the submitted version.

## Conflict of Interest

The authors declare that the research was conducted in the absence of any commercial or financial relationships that could be construed as a potential conflict of interest.
